# Outcomes after long-term mechanical ventilation of cancer patients

**DOI:** 10.1186/s12904-020-00544-x

**Published:** 2020-03-30

**Authors:** Kelly Haviland, Kay See Tan, Nadja Schwenk, Manju V. Pillai, Diane E. Stover, Robert J. Downey

**Affiliations:** 1 Department of Nursing, Memorial Hospital, Memorial Sloan Kettering Cancer Center, New York, NY USA; 2 Department of Epidemiology and Biostatistics, Memorial Sloan Kettering Cancer Center, New York, NY USA; 3 Pulmonary Service, Department of Medicine, Memorial Hospital, Memorial Sloan Kettering Cancer Center, New York, NY USA; 4 Thoracic Service, Department of Surgery, Memorial Hospital, Memorial Sloan Kettering Cancer Center, 1275 York Avenue, New York, NY 10065 USA

**Keywords:** Mechanical ventilation, Ventilation, Weaning, Cancer

## Abstract

**Background:**

The probability of weaning and of long-term survival of chronically mechanically ventilated cancer patients is unknown, with incomplete information available to guide therapeutic decisions. We sought to determine the probability of weaning and overall survival of cancer patients requiring long-term mechanical ventilation in a specialized weaning unit.

**Methods:**

A single-institution retrospective review of patients requiring mechanical ventilation outside of a critical care setting from 2008 to 2012 and from January 1 to December 31, 2018, was performed. Demographic and clinical data were recorded, including cancer specifics, comorbidities, treatments, and outcomes. Overall survival was determined using the Kaplan-Meier approach. Time to weaning was analyzed using the cumulative incidence function, with death considered a competing risk. Prognostic factors were evaluated for use in prospective evaluations of weaning protocols.

**Results:**

Between 2008 and 2012, 122 patients required mechanical ventilation outside of a critical care setting with weaning as a goal of care. The cumulative incidence of weaning after discharge from the intensive care unit was 42% at 21 days, 49% at 30 days, 58% at 60 days, 61% at 90 days, and 61% at 120 days. The median survival was 0.16 years (95% CI, 0.12 to 0.33) for those not weaned and 1.05 years (95% CI, 0.60 to 1.34) for those weaned. Overall survival at 1 year and 2 years was 52 and 32% among those weaned and 16 and 9% among those not weaned. During 2018, 36 patients at our institution required mechanical ventilation outside of a critical care setting, with weaning as a goal of care. Overall, with a median follow-up of 140 days (range, 0–425 days; average, 141 days), 25% of patients requiring long-term mechanical ventilation (9 of 36) are alive.

**Conclusions:**

Cancer patients can be weaned from long-term mechanical ventilation, even after prolonged periods of support. Implementation of a resource-intensive weaning program did not improve rates of successful weaning. No clear time on mechanical ventilation could be identified beyond which weaning was unprecedented. Short-term overall survival for these patients is poor.

## Background

Prolonged mechanical ventilation ranks sixtieth among the reasons for hospitalization but third for total charges generated (US$5 billion in 2005) and first for charges per patient [1]. Patients requiring long-term mechanical ventilatory support are part of a group of patients often referred to as “chronically critically ill,” and they often share other medical conditions, including recurrent infections, neuromuscular dysfunction (due to both disuse and medication), cognitive changes (such as delirium), and pain and other problems. As such, they represent a group of patients with profoundly recalcitrant medical conditions.

The outcomes achieved by medical care of patients requiring mechanical ventilation have been incompletely characterized with regard to the likelihood of both weaning and survival, and even less so with regard to quality of life during the time these patients remain alive. Cancer patients requiring long-term mechanical ventilation have been the subject of only two previous studies. Shih and colleagues reviewed cancer patients who required mechanical ventilation for > 21 days in Taiwan from 1998 to 2007. Half of these patients survived < 1.4 months, and the 1-year overall survival was 14% [2]. In Taiwan, care may not be withdrawn, limiting the application of this work to other populations in which care may be withdrawn if it is believed to be futile. Soares et al. performed a retrospective review of a single institution’s experience with 163 cancer patients requiring > 21 days of mechanical ventilation via tracheostomy in an intensive care unit (ICU), essentially all of whom required mechanical ventilatory support [3]. However, the duration of the need for mechanical ventilation was not provided. The hospital and 6-month survivals were 49 and 40%, respectively, for patients in this study.

In 2010, the Surgical Advanced Care Unit (SACU) was opened at Memorial Hospital, Memorial Sloan Kettering Cancer Center (MSK). Among other clinical programs, the SACU offers a comprehensive program aimed at weaning patients from long-term mechanical ventilation. This weaning program is multidisciplinary (including internists, pulmonologists, specialized NPs, respiratory care, rehabilitation, social work, case management), with a high level of nursing staffing (one RN for two patients) and dedicated physical facilities (rooms designed to accommodate ventilated patients). Since 2010, the SACU staff care for approximately 32 patients each year who require long-term mechanical ventilation.

To measure whether the creation of a dedicated weaning program altered the outcomes seen in this patient population, we performed a single-institution retrospective study of cancer patients requiring long-term mechanical ventilation who were cared for in a specialized intermediate care weaning unit. The goal of this study was to characterize the results achieved, focusing on the likelihood of weaning and on overall survival. Two patient cohorts were examined. The first group was cared for between 2008 and 2011, a period that was chosen so that a comparison between patients receiving care before and after creation of a dedicated weaning unit could be performed. The second group was cared for between January and December 2018, a period chosen because it represents contemporary practice and provides sufficient time for clinical follow-up to estimate long-term outcomes.

## Materials and methods

### Design, setting, and eligibility criteria

After a waiver of authorization (WA0023–13) was obtained from the Institutional Review Board at Memorial Sloan Kettering Cancer Center, we performed a retrospective review of a single institution’s experience with all patients treated with prolonged mechanical ventilation with weaning as a goal of care after ICU discharge, subject to intensivist discretion, between 2008 and 2012 and between January and December 2018. Pediatric (< 18 years of age) and neurological patients were not included. During the initial 2 years of the study period (2008–2009), the primary responsibility for care of patients treated with prolonged mechanical ventilation after ICU discharge was by the service that had initially admitted the patient to the hospital on their primary floor. In 2010, the surgical advanced care unit (SACU) was created. Under the SACU program, all patients treated with prolonged mechanical ventilation after discharge from the ICU were transferred to the care of the General Medicine Service. A coordinated program of care was delivered, organized around daily rounds attended by the SACU nurse practitioners and registered nurses, the General Medicine attending, and the Pulmonary Medicine attending, as well as representatives from the previous primary service, Respiratory Therapy, Physical Therapy, Occupational Therapy, Social Work, and Case Management.

### Data collection and processing

Demographic and clinical data were recorded, including cancer specifics, comorbidities, treatments, and outcomes. The data fields collected and definitions used are listed in the Supplemental Materials. Weaning as a goal of care was determined from a subjective review of the daily determinations made by the treating team of attending physicians. Successful weaning was defined as removal from all mechanical ventilatory support for 48 h.

### Statistical methods

Patient demographic and clinical characteristics were summarized using descriptive statistics. By use of the Kaplan-Meier method, overall survival was calculated from the time of ICU discharge until the date of death or was censored on the date of last clinical contact. Weaning status was treated as a time-dependent covariate: all patients began as “not weaned” upon ICU discharge, and the weaning status changed to “weaned” on the date of first documentation of weaning, if applicable. Comparisons of overall survival by group were made using the log-rank test. Cox regression was used for univariable and multivariable analyses to estimate the hazard of death based on weaning statuses, with adjustment for demographic and clinical variables. Factors included care in the SACU, age on initiation of ventilatory support outside of the ICU, sex, cancer status (active versus no evidence of disease), tumor histology (solid versus liquid), and number of days on mechanical ventilation while in the ICU. Time to weaning, defined as the number of months between ICU discharge and first documentation of successful weaning, was analyzed using a competing risks approach. Death without having been weaned was treated as a competing risk event. Cumulative incidence functions for each competing event were calculated using competing risks methodology [4]. Fine and Gray’s competing risk regressions for the sub-hazard ratio [5] were used to evaluate any patient, tumor, or treatment characteristic that was associated with the incidence of weaning. We derived the conditional probability of weaning in the presence of a competing risk (death without having been weaned) in a period, assuming the surviving patient was not yet weaned at the beginning of the period [6]. Analyses were conducted using R 3.1.1 (R Development Core Team, Vienna, Austria) and Stata 13 (StataCorp, College Station, TX). All statistical tests were 2-sided, and *P* < 0.05 was considered to indicate statistical significance.

## Results

Between January 1, 2008, and December 31, 2012, 181 patients were mechanically ventilated at Memorial Hospital outside of a critical care unit (excluding pediatric and neurological patients, who were treated in separate, specialized programs). Of the 181 patients, 130 were treated with weaning as a goal of care; the remainder were treated with palliative intent. For the purposes of this study, to achieve uniformity in the study populations, 8 patients who had not been admitted to the ICU were excluded from the analyses, resulting in 122 patients included in the study cohort.

The demographic and clinical characteristics of the 122 patients with weaning as a goal of care are summarized in Table 1. In total, 91% of the patients either had known active malignancies or were being actively treated with antineoplastic therapy. A majority of the patients with cancer (90%) had solid tumors. The median follow-up for this cohort was 4.0 months (range, < 1 month to 6.7 years); for patients in this cohort who died, the median follow-up was 3.2 months (range, < 1 month to 3.6 years).

**Table 1:** Patient Characteristics (*N* = 122)

Characteristic	Total
Care in SACU	
No	47 (39)
Yes	75 (61)
Sex	
Female	56 (46)
Male	66 (54)
Cancer status	
Active	111 (91)
No evidence of disease	11 (9)
COPD (*N* = 26)	
No	20 (77)
Yes	6 (23)
Cancer histologic profile (*N* = 120)	
Liquid tumor	12 (10)
Solid tumor	108 (90)
Age, years	69.0 (55.0 to 76.0)
ICU and mechanical ventilator duration	
ICU days (*N* = 121)	20.0 (13.0 to 27.0)
ICU ventilation days (*N* = 121)	18.0 (10.0 to 25.0)
Days on ventilation outside ICU	13.5 (8.0 to 34.0)
Total ventilation days while inpatient	34.0 (24.0 to 54.0)
Weaned	
No	60 (49)
Yes	62 (51)
Died while on mechanical ventilation (*N* = 99)	
No	64 (65)
Yes	35 (35)
Deceased	
No	21 (17)
Yes	101 (83)
Discharged on ventilation (*N* = 6), suspected dead	6 (100)

Data are no. (%) or median (25th to 75th percentile)

*COPD* chronic obstructive pulmonary disease, *ICU* intensive care unit, *SACU* surgical advanced care unit

**Fig. 1:**
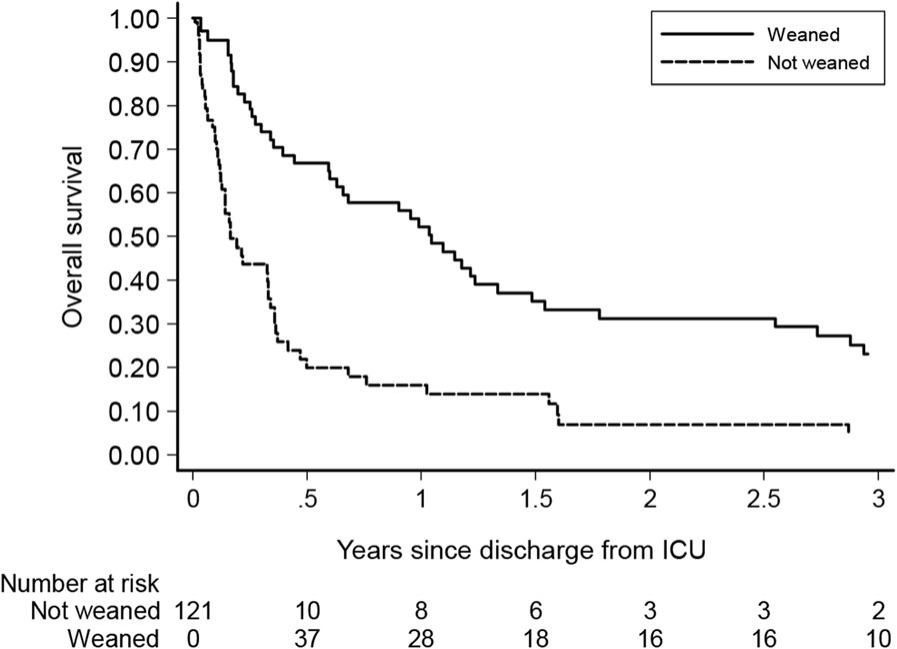
Overall survival of weaned and not weaned cancer patients. Weaning status was entered as a time-dependent covariate. All patients were discharged from ICU while on mechanical ventilation and thus were not weaned at ICU discharge; the patient’s status was changed to “weaned” at the date of the first recorded weaning

Of the 122 patients in the study cohort, 62 (51%) were weaned from mechanical ventilation. Of all the cancer patients with weaning as a goal of care and requiring 14 days of mechanical ventilation after ICU discharge, 43% were eventually weaned. Of patients requiring 28 days of mechanical ventilation after ICU discharge, 23% were eventually weaned (Table 2). Figure 1 shows the cumulative incidence of weaning since ICU discharge, along with the curve for the competing risk of death without weaning. The median (25th to 75th percentile) duration on ventilators since ICU discharge, for patients who were weaned, was 10 (5 to 18) days; for those who were not weaned, this was 24 (11 to 49) days. The probability of a surviving patient being weaned by a certain time (i.e., if the patient had neither died nor been weaned) after ICU discharge was 35% at 14 days, 41% at 21 days, and 56% at 60 days (Table 2).

**Table 2:** Cumulative Incidence of Being Weaned by Selected Time Points after ICU discharge

Time Point	Cumulative Incidence (95% CI)
14 days	0.35 (0.26 to 0.43)
21 days	0.41 (0.32 to 0.50)
30 days	0.46 (0.37 to 0.55)
60 days	0.56 (0.45 to 0.65)

*CI* confidence interval

Patients who either had active disease or were receiving antineoplastic therapy were approximately 47% less likely to be weaned at any given time point, compared with patients who did not have active disease or who were not undergoing treatment (Table 3). Patients with solid tumors were not significantly more likely to be weaned than patients with liquid tumors (*P* = 0.3).

**Table 3:** Univariable Competing Risk Regression of Weaning from the Date of ICU Discharge (SHR) and Univariable Cox Model of Hazard of Death from date of ICU discharge (HR)

Variable	HR (95% CI)	*P*	SHR (95% CI)	*P*
Weaned^a^	0.37 (0.24 to 0.57)	< 0.0001	n/a	n/a
Care in SACU	0.96 (0.65 to 1.43)	0.9	0.79 (0.48 to 1.31)	0.4
Sex—male	1.23 (0.83 to 1.82)	0.3	0.91 (0.55 to 1.51)	0.7
Cancer status—active disease	1.70 (0.83 to 3.52)	0.15	0.47 (0.33 to 0.67)	< 0.0001
Solid—yes	0.61 (0.33 to 1.13)	0.12	1.80 (0.62 to 5.24)	0.3
Age	0.99 (0.98 to 1.01)	0.4	1.00 (0.99 to 1.02)	0.7
Number of ICU days on MV	1.00 (0.98 to 1.01)	0.6	1.00 (0.98 to 1.01)	0.5
Days on ventilation in ICU > 21	n/a		0.89 (0.54 to 1.49)	0.7

*CI* confidence interval, *ICU* intensive care unit, *SACU* surgical advanced care unit, *SHR* subdistribution hazard ratio, *MV* mechanical ventilation, *HR* hazard ratio

^a^Time-varying covariate: weaning status changed from “not weaned” to “weaned” on the date of first documentation of weaning post–ICU discharge

**Fig. 2:**
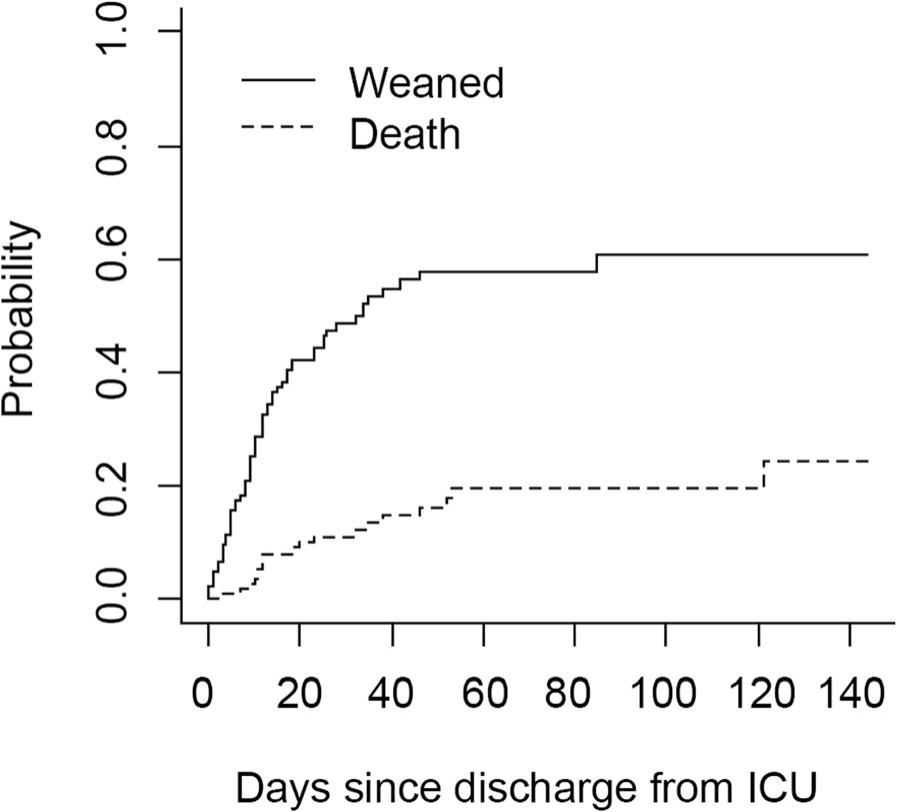
The cumulative incidence of weaning after ICU discharge and of the competing risk of death without weaning. The cumulative incidence of weaning was 42% at 21 days after ICU discharge

The median survival for patients who had weaning as a goal of care but who were not weaned was 0.16 years (95% CI, 0.12 to 0.33), compared with 1.05 years for those who were weaned (95% CI, 0.60 to 1.34). The 1-year overall survival for patients who were not weaned was 16% (95% CI, 8 to 27%), compared with 52% for patients who were weaned (95% CI, 38 to 64%) (*P* < 0.001) (Figs. 1 and 2). The 2-year overall survival for patients who were weaned was 32% and for patients who were not weaned was 9%. When weaning status was used as a time-varying covariate, the hazard of death for patients who were able to be weaned was 0.37 times that for those who were not able to be weaned (95% CI, 0.24 to 0.57) (*P* < .001); conversely, the hazard of death for patients who were not weaned was 2.67 times higher than that for patients who were successfully weaned (95% CI, 1.74 to 4.09) (*P* < 0.001). Among the univariable Cox proportional hazards models, weaning status was the only factor that was significantly associated with overall survival (Table 3), so a multivariable analysis was not performed.

The second cohort of patients examined were cared for between January 1 and December 31, 2018. During this interval, there were 36 unique patients admitted to SACU for ventilator management. Six of 36 patients were admitted to SACU more than once. The median hospital length of stay (LOS) for the patients was 58 days (average, 72 days; range, 14–212 days). The median SACU LOS for all patients was 9 days (average, 21; range, 1–122 days). Thirty percent of patients who were in the SACU on ventilator for > 21 days were subsequently able to be weaned.

The discharge status of the 36 patients was as follows. Thirty-eight percent of patients (14 of 36) were discharged alive from the hospital. Eight percent of patients (3 of 36) were discharged to home. Thirty percent of patients (11 of 36) were discharged to a long-term care facility or hospice. Sixty-one percent of patients (22 of 36) died while inpatients at MSK. At last follow-up, the status of 14 patients discharged from MSK was 3 to home, 3 to hospice, 7 to a rehabilitation facility, and 1 unknown from chart review. The most frequent disposition of patients with SACU LOS ≥21 days (13 patients) was death at MSK (8 patients). Of the 5 patients with SACU LOS ≥21 days, 4 patients were weaned and 1 was not weaned.

Overall, with a median follow-up of 140 days (range, 0–425 days; average, 141 days), 25% of patients requiring long-term mechanical ventilation at MSK (9 of 36) are alive.

## Discussion

We found that the 1-year and 2-year overall survival for patients who were weaned were 52 and 32%. The 1-year and 2-year overall survival for patients who were not weaned were 16 and 9%. Again, the 1-year overall survival for weaned patients in our study was similar to survival rates in previous reports including non-cancer patients. Engoren et al. [7], in 2004, reported a 1-year overall survival of 58%. Bigatello [8], in 2007, reported a similar 1-year overall survival, of 61%. Similar outcomes have been reported in more recent studies: in a retrospective multicenter review from 2012, Carson et al. [9] found a 1-year mortality of 48%. Unfortunately, there are few reports documenting 2-year survival outcomes to compare our results with.

Efforts to improve long-term outcomes after prolonged mechanical ventilation have been reported. Daly et al. [10] reported a prospective randomized trial of patients cared for either by a disease management team (DMT)—including nurse practitioners, a geriatrician, and a pulmonologist—or by the primary service alone. Care by the DMT extended to 2 months after hospital discharge. Unfortunately, neither mortality, need for rehospitalization, nor time to rehospitalization was significantly improved in the DMT care group. This emphasizes the recalcitrance and intractability of the medical problems experienced by this patient population. Similarly, for the patients that compose our study population, at first, care was directed by the original admitting service, with weaning directed by the Critical Service as a consult service. In 2010, we created the SACU, which consolidated all patients requiring mechanical ventilation outside of an ICU to one floor with dedicated ventilator rooms, including monitoring, and a cadre of nurse practitioners who were physically present on the floor 24–7. All mechanically ventilated patients were transferred to the General Medicine Service, which coordinated care during daily rounds (7 days a week) with representatives from nursing (both registered nurses and SACU-dedicated nurse practitioners) as well as Pulmonary Medicine, Respiratory Therapy, Rehabilitation Medicine (i.e., Physical and Occupational Therapy), and Social Work Services. The outcomes of patients cared for in the SACU, despite this coordination of care and expenditure of resources, were not significantly different from the outcomes of patients cared for before the creation of the SACU. It is possible that our weaning program was more effective than our data suggest because of other factors that are difficult to account for. For example, during this same time, the Memorial Hospital ICU implemented multiple other care plans directed at improving the likelihood of weaning while in the ICU, including sedation holidays [11] and mobilization while on mechanical ventilatory support [12]. It is likely that these programs led to earlier successful weaning of more patients while they were admitted to the ICU and that the remaining patients, who were discharged from the ICU to SACU care while on mechanical ventilation, represented a sicker patient population with more recalcitrant medical conditions.

Our study provides information on the likelihood of weaning and on survival for cancer patients requiring long-term mechanical ventilation. It is our hope that this information can be provided to patients and their families to assist in clinical decision-making. Specifically, with the goal of guiding care in mind, we examined whether an inflection point for success in weaning associated with the duration of weaning might be found. For example, an early period characterized by a high success rate in weaning might be followed by a plateau in weaning success, such that transition to a care plan for long-term ventilatory support could be made. No such inflection point seems apparent, and if it does exist, it seems likely to occur between 45 and 60 days (Fig. 2).

Our study also does not provide what may be more important information: the likely quality of life during a patient’s remaining life. Multiple studies have noted diminished quality of life [7, 13–15], including persistent significant cognitive deficiencies [13, 16], after care that included prolong mechanical ventilation. In general, these outcomes are not anticipated by patients and their caregivers. Cox et al. [17] found that, at the time of initiation of ventilation, 93% of patients and caregivers expected the patient to be alive at 1 year, 71% expected good functional status, and 83% expected a good quality of life. After a year, only 56% of patients were alive, and of these survivors only 9% had a good functional status and 33% had a good quality of life. Taken together, these findings suggest that palliative care teams should be included in the overall management of patients requiring long-term mechanical ventilation [18].

Future research could include collecting detailed information about patient quality of life and then examining whether providing patients and their families detailed information about the quality and duration of life that is likely to be experienced by a patient alters the clinical decisions patients and families make.

## Supplementary information

**Additional file 1.**

## Data Availability

The datasets used and/or analysed during the current study are available from the corresponding author on reasonable request.

## Abbreviations

DMT: Disease management team
ICU: Intensive care unit
SACU: Surgical advanced care unit
