# Supplementary material for: Outcomes after long-term mechanical ventilation of cancer patients
Source: BMC Palliat Care. 2020 Mar 30;19:42. doi: 10.1186/s12904-020-00544-x (PMC7106688; doi:10.1186/s12904-020-00544-x)
Supplement: Supplementary file 1 — Additional file 1. [file 12904_2020_544_MOESM1_ESM.docx]

**Supplemental materials**

**Patient demographics and clinical characteristics collected:**

- Age
- Sex
- Cancer characteristics: Histologic profile, stage at diagnosis, location, current disease status
- Histologic profile was categorized as liquid tumor, solid tumor, thoracic, hepatopancreaticobiliary, colorectal, gastric/mixed tumor, gynecologic, head and neck, urologic, orthopedic, breast, lymphoma, or leukemia
- Cancer disease status was categorized as alive with disease or no evidence of disease.
- Acute physiology and chronic health evaluation (APACHE IV) and mortality score
- Noncancer-related medical characteristics: Asthma, chronic obstructive pulmonary disease, and ejection fraction upon admission, comorbidities, smoking history,
- Laboratory values: Hemoglobin, albumin, carbon dioxide

Clinical characteristics: Weaning as a goal of care, date of intubation, date of tracheostomy, date admitted to the ICU, number of ICU days, date of transfer from the ICU to the SACU or floor, resuscitation status (DNR vs. to be resuscitated), date made DNR date, total number of days on the ventilator, first ventilator day, last ventilator day, number of days on the ventilator outside of the ICU, admitted to surgical or medical service, date of wean from mechanical ventilation, date of decannulation, date of discharge, discharge location, date of death or other last known clinical contact
